# Amplicon Sequencing-Based Bipartite Network Analysis Confirms a High Degree of Specialization and Modularity for Fungi and Prokaryotes in Deadwood

**DOI:** 10.1128/mSphere.00856-20

**Published:** 2021-01-13

**Authors:** Julia Moll, Anna Heintz-Buschart, Claus Bässler, Martin Hofrichter, Harald Kellner, François Buscot, Björn Hoppe

**Affiliations:** a Department of Soil Ecology, UFZ - Helmholtz Centre for Environmental Research, Halle (Saale), Germany; b German Centre for Integrative Biodiversity Research (iDiv), Halle-Jena-Leipzig, Germany; c Department of Biodiversity Conservation, Institute for Ecology, Evolution and Diversity, Faculty of Biological Sciences, Goethe University Frankfurt, Frankfurt am Main, Germany; d Bavarian Forest National Park, Grafenau, Germany; e Department of Bio- and Environmental Sciences, Technische Universität Dresden - International Institute (IHI), Zittau, Germany; f Institute for National and International Plant Health, Julius Kühn-Institut, Braunschweig, Germany; University of Wisconsin—Madison

**Keywords:** amplicon sequencing, bipartite networks, deadwood, decomposition, microbes, modularity, specialization

## Abstract

Fungi and prokaryotes are dominant colonizers of wood and mediate its decomposition. Much progress has been achieved to unravel these communities and link them to specific wood properties. However, comparative studies considering both groups of organisms and assessing their relationships to wood resources are largely missing. Bipartite interaction networks provide an opportunity to investigate this colonizer-resource relationship more in detail and aim to directly compare results between different biotic groups. The main questions were as follows. Are network structures reflecting the trophic relationship between fungal and prokaryotic colonizers and their resources? If so, do they reflect the critical role of these groups, especially that of fungi, during decomposition? We used amplicon sequencing data to analyze fungal and prokaryotic interaction networks from deadwood of 13 temperate tree species at an early to middle stage of decomposition. Several diversity- and specialization-related indices were determined and the observed network structures were related to intrinsic wood traits. We hypothesized nonrandom bipartite networks for both groups and a higher degree of specialization for fungi, as they are the key players in wood decomposition. The results reveal highly modular and specialized interaction networks for both groups of organisms, demonstrating that many fungi and prokaryotes are resource-specific colonizers. However, as the level of specialization of fungi significantly surpassed that of prokaryotes, our findings reflect the strong association between fungi and their host. Our novel approach shows that the application of bipartite interaction networks is a useful tool to explore, quantify, and compare the deadwood-colonizers relationship based on sequencing data.

**IMPORTANCE** Deadwood is important for our forest ecosystems. It feeds and houses many organisms, e.g., fungi and prokaryotes, with many different species contributing to its decomposition and nutrient cycling. The aim of this study was to explore and quantify the relationship between these two main wood-inhabiting organism groups and their corresponding host trees. Two independent DNA-based amplicon sequencing data sets (fungi and prokaryotes) were analyzed via bipartite interaction networks. The links in the networks represent the interactions between the deadwood colonizers and their deadwood hosts. The networks allowed us to analyze whether many colonizing species interact mostly with a restricted number of deadwood tree species, so-called specialization. Our results demonstrate that many prokaryotes and fungi are resource-specific colonizers. The direct comparison between both groups revealed significantly higher specialization values for fungi, emphasizing their strong association to respective host trees, which reflects their dominant role in exploiting this resource.

## Abstract (precis)

Deadwood is important for our forest ecosystems. It feeds and houses many organisms, e.g., fungi and prokaryotes, with many different species contributing to its decomposition and nutrient cycling.

## INTRODUCTION

Ecological interaction networks have become widely used tools to investigate the organization of interacting organisms at the community level. Analysis of these networks provides the opportunity to explicitly explore communities of interest by the comparison of network topologies in relation to relevant environmental properties. Much progress has been made on bipartite networks investigating two species’ groups, mostly from two trophic levels, that interact with each other. This approach has been used to elucidate relevant ecological relationships, e.g., plant-pollinator, plant-microbe, or host-parasitoid interactions (1–4). In order to assess the structure of such networks precisely, several indices have been developed. Those can be calculated at the species level, resulting in one value for each species (e.g., effective number of partners); at the group level, resulting in one value for each of the two groups (e.g., generality: mean number of partners per group); and at the network level, revealing one value for the entire network (e.g., mean number of realized links) (5). For the latter, the identification and quantification of indices such as Shannon and interaction evenness commonly describe the diversity of a network. Other indices at the network level have been developed to describe the degree of specialization (5, 6). Bipartite networks often consist of several subcommunities that are clustered on a subset of hosts or resources. Organisms within such “modules” interact more among each other than with the rest of the network. Indices such as modularity and *H_2_′* represent estimators for a modular structure and provide the opportunity to compare the degree of specialization between networks based on different sources, as both values are largely independent of matrix size and sampling effort (7, 8).

This comparability facilitates the exploration of specific biotic groups under various environmental conditions and regions, e.g., plant-fungal interactions in relation to successional plant stage or tree diversity level or across elevational gradients (9–11). Network analyses, in turn, can be used to draw conclusions on ecosystem stability, as highly connected and less-specialized networks are assumed to be more robust to disturbances such as drought or, more generally, climate change (8, 12, 13). Moreover, this approach ensures the standardized comparison of different ecological groups, e.g., guilds, under similar conditions (14, 15). It turned out that the organization of networks is related to the type of the underlying trophic relationship; mutualistic networks appear to be highly nested and specialized, whereas antagonistic networks are specialized and highly modular (16).

Recently, bipartite network analyses have been applied in forest ecology to explore the trophic relationship of wood-colonizing organisms to their deadwood resource (17–19). Deadwood represents an important substrate in forest ecosystems that contributes to nutrient cycling, acts as carbon storage, and provides habitat for many saproxylic organisms. Fungi are among the key wood-colonizing species, as are prokaryotes, which are able to degrade various plant-derived carbon resources and thus mediate intermediary steps in the decomposition of deadwood (20). Fungi contribute significantly to this ecosystem process through the incipient attack on recalcitrant lignin and associated cell wall polysaccharides (21). This capacity is mainly restricted to basidiomycetes and xylariaceous ascomycetes that produce an effective array of extracellular oxidoreductases and hydrolytic enzymes (22–24). Some wood-colonizing prokaryotes are also capable of degrading cellulose and hemicelluloses, and their contribution to lignin degradation or its chemical modification in deadwood is currently under discussion (25). Besides prokaryotes that were identified to actively degrade these wood components, others simply live from wood and fungal residues or in tertiary links to insects without contributing to wood decay (25). However, several of these microbes may indirectly contribute to wood decomposition by making the wood more permeable or as synergists that stimulate other degrading organisms (26). For instance, the ability of bacteria such as *Rhizobiales* to fix nitrogen (N_2_) from the atmosphere is essential for other saproxylic organisms in the N-limited environment of deadwood, resulting in a stimulating effect for other biota (27).

The current study presents findings from the BELongDead (Biodiversity Exploratories Long-term Deadwood) experiment that observes decomposition of deadwood logs of 13 deciduous and coniferous temperate tree species, standardized by the same starting time point of decomposition (28). Prior to this study, the wood-colonizing fungal and prokaryotic communities and their spatial distribution in sapwood and heartwood were analyzed using amplicon sequencing, revealing tree species-related differences for both groups and spatial differences mainly for the prokaryotes (29, 30). However, it remained unclear how the two groups are comparatively linked to the deadwood resource.

By reconstructing bipartite interaction networks and calculating network statistics for these fungal and prokaryotic data sets, we aimed at pursuing this question to resolve the colonizer-resource relationship, as this approach allows a direct comparison between the two groups. The main research question was whether network structures reflect the trophic relationship between colonizers and wood resources. The broad phylogenetic range of the investigated deadwood from the 13 tree species represented a resource distinguishable by a wide set of environmental variables, e.g., various physicochemical properties, which deadwood-colonizing organisms were exposed to (compare T. Kahl et al. [28]).

Specifically, we tested the following hypotheses. (i) As initiators and main drivers of wood decomposition, fungi are organized in networks that are highly specialized. (ii) As prominent deadwood-colonizing organisms and degraders of several plant materials, prokaryotic networks are specialized, but to a lesser extent than fungal networks. (iii) As heartwood-colonizing organisms are exposed to specific habitat conditions, e.g., a larger amount of extractives and/or lower levels of dioxygen, the topology of sapwood and heartwood networks differ for the two groups of organisms.

## RESULTS

**FIG 1:**
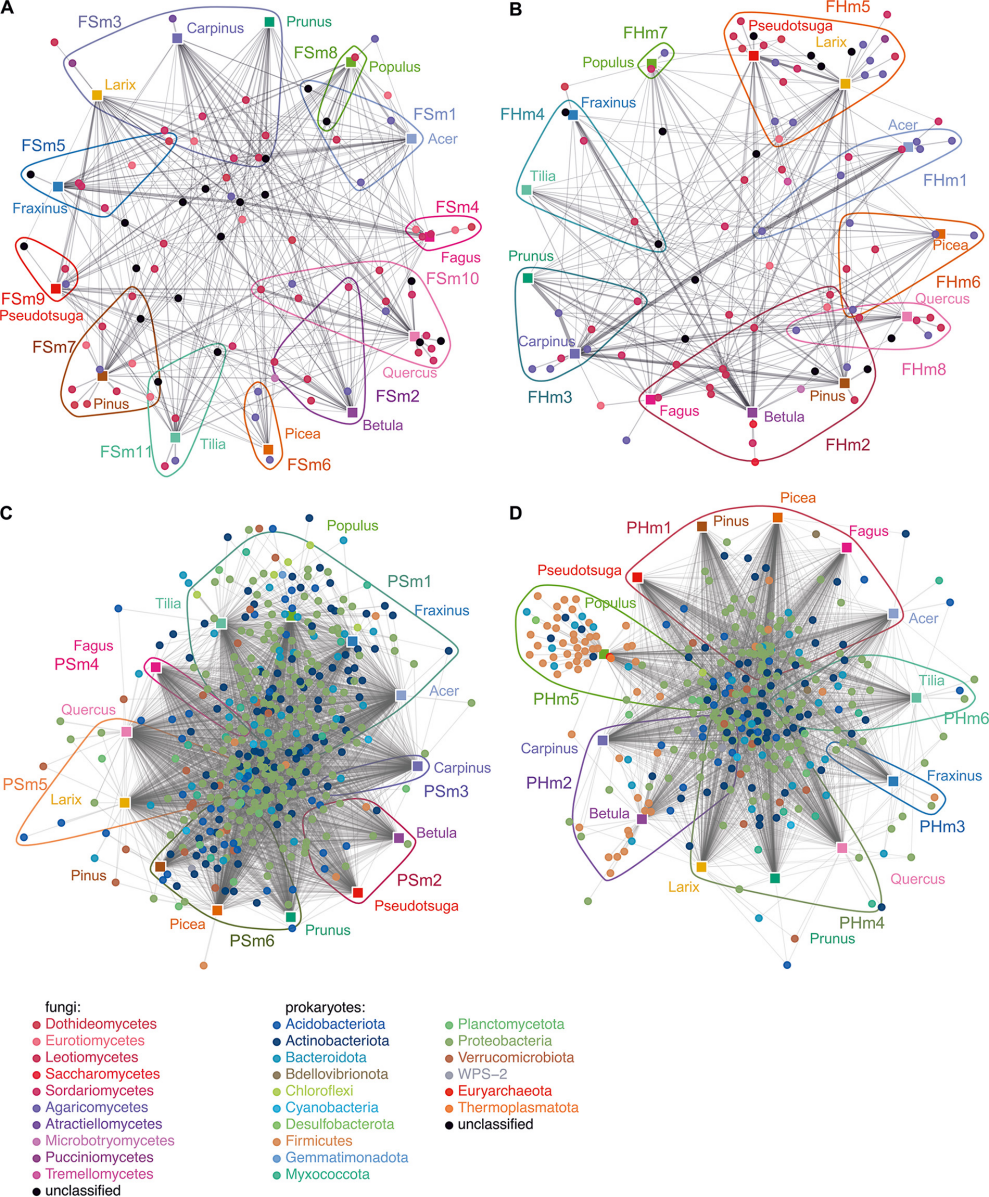
Bipartite networks for the fungal (A and B) and prokaryotic (C and D) colonizers of the sapwood (A and C) and heartwood (B and D) of the deadwood of 13 tree species. Each panel shows a visual representation of OTUs (●) colored according to fungal classes and prokaryotic phyla that were present in all 1,000 rarefactions. Modules are indicated around OTUs that were consistently associated with the respective member trees (■). The figure demonstrates the modular structure of networks and especially the high number of modules for the fungal networks. Module-associated trees and OTUs and their relative abundances and identities are given in Tables S2 to S5. FSm, fungal sapwood module; FHm, fungal heartwood module; PSm, prokaryotic sapwood module; PHm, prokaryotic heartwood module.

Bipartite network analyses of rarefied data were performed based on 1,878,668 sequences representing 2,700 fungal operational taxonomic units (OTUs) and 1,851,687 sequences representing 10,849 prokaryotic OTUs from deadwood logs of 13 temperate tree species (3 replicates each). For 1,000 rarefied versions, network structures for both groups of organisms and wood compartments differed from those of their respective null models. Generally, more prokaryotic OTUs than fungal OTUs were included in the sapwood and heartwood networks. Network size, on average, included 233 and 207 fungal OTUs and 1,412 and 1,097 prokaryotic OTUs for sapwood- and heartwood-based networks, respectively. Ten fungal classes all belonging to Dikarya (Ascomycota and Basidiomycota) were consistently identified in all 1,000 rarefaction versions, of which basidiomycetous Agaricomycetes and ascomycetous Sordariomycetes were the most dominant classes (Tables S2 and S3; Fig. 1A and B). The prokaryotic interaction networks were consistently formed by OTUs belonging to 16 phyla, with *Proteobacteria* (*alpha*- and *gammaproteobacteria*) being the most abundant bacterial and *Euryarchaeota* (*Methanobacteria*) the most abundant archaeal phylum (Tables S4 and S5; Fig. 1C and D). Based on the 13 tree species investigated, 11 distinct modules were detected within the sapwood-based fungal network, of which only one module (fungal sapwood module 3 [FSm3]: *Carpinus*, *Larix*, *Prunus*) consisted of more than one deadwood tree species (Fig. 1A). The heartwood-based fungal network was organized into eight modules (Fig. 1B). Although both prokaryotic networks revealed six modules each, tree species were not grouped into the same modules in the sapwood and heartwood networks (Fig. 1C and D). Similar results were observed at different rarefication depths, after exclusion of rare OTUs (up to tripletons), and based on networks reconstructed from samples of single plots.

**FIG 2:**
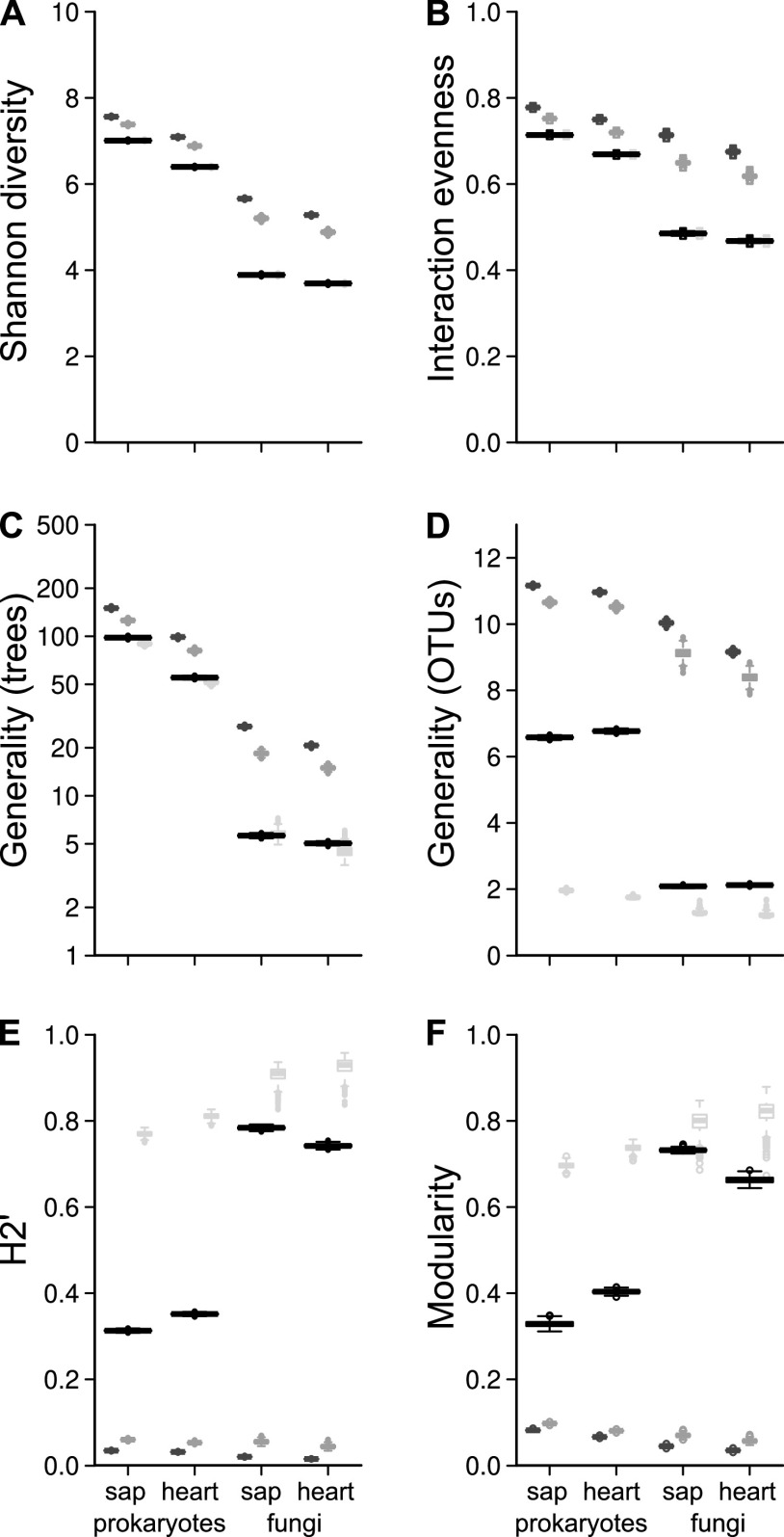
Comparison of network indices associated with prokaryotic and fungal colonizers of the sapwood and heartwood of the deadwood of 13 tree species. (A) Shannon diversity of network entries. (B) Interaction evenness (Shannon’s evenness of network entries). (C) Generality of trees, weighted mean effective number of associated OTUs per tree species. (D) Generality of OTUs, weighted mean effective number of associated trees per fungal or prokaryotic OTU; higher values indicate more general interactions. (E) *H_2_′* based on the deviation of a species’ realized number of interactions and that expected from each species’ total number of interactions (ranges between 0, no specialization and 1, perfect specialization). (F) Modularity, bipartite algorithm of Newman’s modularity (ranges between 0, no modularity and 1, perfect modules). Smaller gray boxplots represent results of respective null models (left to right: dark gray, Patefield; gray, Vazquez; light gray, shuffle [the latter displaying similar values as the observed networks in panels A and B]).

All estimated network indices differed significantly from the null models (*P* values < 0.00001), except for the Shuffle null model (Fig. 2 and Table S6). For the two diversity indices, Shannon and interaction evenness, no differences were expected, as the Null model’s connectance has to be equal to the observed data. Significant differences between fungal and prokaryotic networks were corroborated by comparison of plot-wise networks, whereas the comparison between sapwood and heartwood within the fungal and prokaryotic data sets revealed only significantly different values for prokaryotic generality of trees (Table S7). Shannon diversity and interaction evenness were highest for the prokaryotic sapwood-based network, while the lowest values were detected for the fungal heartwood network (Fig. 2A and B). Generality of trees (mean number of associated OTUs per tree) and generality of OTUs (mean number of tree species per OTU) were significantly higher for both prokaryotic interaction networks than for the fungal networks (*P* values ≤ 0.01; Fig. 2C and D and Table S7). In particular, the generality of trees was, on average, 5 in the fungal networks compared to 55 (heartwood) and 98 (sapwood) in the prokaryotic networks. In contrast, generality of fungal OTUs was 2.1 on average but 6.6 (sapwood) and 6.8 (heartwood) for prokaryotic OTUs (Fig. 2D). Generally, indices related to specialization were high for both wood-colonizing groups. Trees were significantly more specialized for fungal interaction partners than for interactions with prokaryotes in both the sapwood and heartwood networks (Blüthgen’s d; *P* values < 0.00001; Tables S8 and S9). The fungal sapwood-based network had the highest mean *H_2_′* value (0.78), followed by the respective heartwood network (0.74) (Fig. 2E). In contrast, for the prokaryotic interaction networks, *H_2_′* had a higher value for the heartwood (0.35) than for sapwood (0.31). Modularity displayed a similar pattern, with the highest value (0.73) for the fungal sapwood-based network and the lowest (0.33) for the prokaryotic sapwood-based network (Fig. 2F).

**FIG 3:**
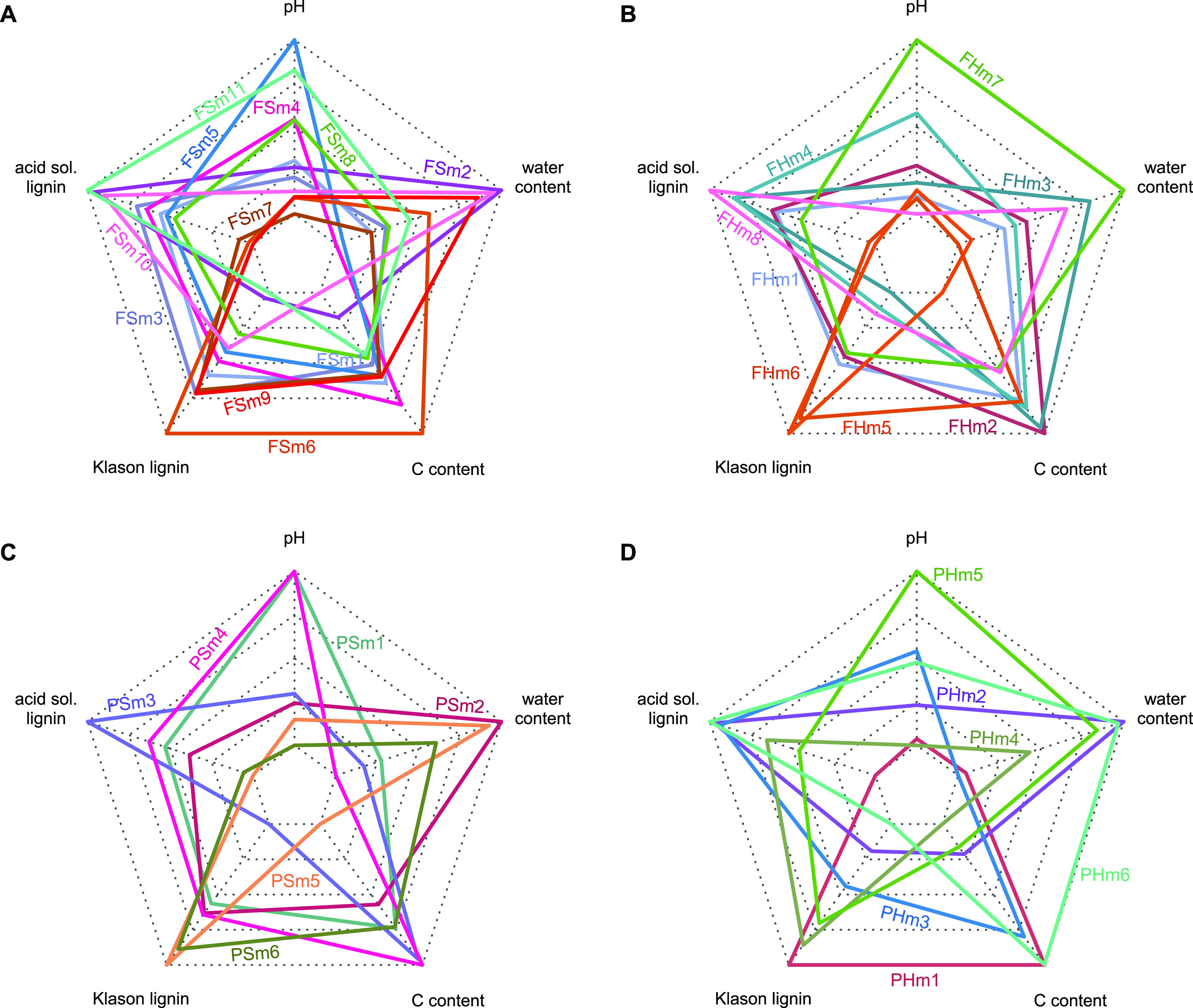
Radar charts illustrating the relationship (min-max scale) between the modules obtained and wood traits (pH, water content, carbon content, Klason lignin, and acid-soluble lignin) for fungal (A and B) and prokaryotic (C and D) networks in the sapwood (A and C) and heartwood (B and D) of the deadwood of 13 tree species.

Wood traits differed significantly between the heartwood of different tree species for lignin and acid-soluble lignin, water content, pH, and C content, and between the sapwoods for acid-soluble lignin (30). Modules of the sapwood fungal network were formed on the basis of tree identity rather than wood traits. This network formed only one module of similarly colonized tree species (FSm3: *Carpinus*, *Larix*, *Prunus*), which was associated with an intermediate pH and variable lignin content (Fig. 3A). In the fungal heartwood-based network, modules differed with respect to acid-soluble lignin and pH (Fig. 3B; Fig. S1A and D). For instance, a high number of specific OTUs was observed in fungal heartwood module 5 (FHm5: *Larix*, *Pseudotsuga*), which was characterized by low acid-soluble lignin content (Fig. 1B, Fig. S1D, Tab. S3). The sapwood-based prokaryotic network formed several large, interconnected modules (Fig. 1C). While these modules differed more strongly in pH and water content than the individual sapwood tree species, the wood traits could not well explain the modularization, indicating that other factors contribute to defining the community assembly in the sapwood. In contrast, the amount of Klason lignin, water content, and the pH value together explained the observed modules in the heartwood prokaryotic network (Fig. 3D; Fig. S1). Specifically, large differences in pH were observed between modules of the heartwood networks (Fig. 3D; Fig. S1A). For instance, in the prokaryotic heartwood-based network, the module with the highest observed pH value (prokaryotic heartwood module 5 [PHm5]: *Populus*) exhibited the highest degree of specialization (*d′* = 0.71) (Fig. 1D; Tab. S8).

10.1128/mSphere.00856-20.1 FIG S1 (A) pH value and content of (B) water, (C) Klason lignin, (D) acid-soluble lignin, and (E) carbon for the modules formed by respective member trees of the fungal sapwood network, fungal heartwood network, prokaryotic sapwood network, and prokaryotic heartwood network (left to right). FSm, fungal sapwood module; FHm, fungal heartwood module; PSm, prokaryotic sapwood module; PHm, prokaryotic heartwood module. Download FIG S1, PDF file, 0.1 MB. Copyright © 2021 Moll et al. 2021 Moll et al. This content is distributed under the terms of the Creative Commons Attribution 4.0 International license.

## DISCUSSION

In this study, we explored bipartite interaction networks and related topologies to better understand the colonizer-resource relationship for two relevant decomposer groups in deadwood. It has been recently shown that there are distinct communities of wood-inhabiting organisms associated with deadwood resources represented by 13 tree species (29–31). As the distribution and abundance of taxa contribute to the structure of ecological networks (32), nonrandom bipartite networks could be anticipated. Therefore, to our best knowledge, a quantitative comparison between fungal and prokaryotic community organization in deadwood is still lacking, and the present study demonstrates strong differences between the two groups.

In line with hypothesis 1, a high level of specialization was observed for both investigated groups, with the fungal networks far surpassing their prokaryotic counterparts. This was reflected in lower values of the generality of trees and fungal OTUs, indicating higher specialization at the group level. Higher values of *H_2_′* and modularity demonstrate the modular structure of the fungal networks being composed of several subcommunities. Indeed, for the fungal sapwood network almost all tree species formed their own module, the exceptions being *Larix*, *Prunus*, and *Carpinus* (FSm3). Despite strong variation in the wood traits of these broadleaved and coniferous tree species, they were grouped together, mainly based on the presence of ascomycetous OTUs, especially some dominant *Helotiales* (e.g., *Leptodontidium* sp.) (Table S2), which could not be classified on a higher taxonomic level. The extent to which these fungi contribute to wood decomposition is difficult to evaluate, as this fungal order is functionally highly diverse, including endophytes and opportunistic saprotrophs (with a mold-like lifestyle) but also soft-rot fungi (33, 34). A broader host selection for *Helotiales* was also observed for fruiting bodies during a citizen science-based data acquisition on 91 woody plant genera in Denmark (35). The authors observed impacts of host tree phylogeny on network modularity and identified wood traits as main driving factors for interactions between fungal fruiting bodies and deadwood hosts. Even though we observed some tree species of similar wood traits that were grouped into one module in the heartwood network, such as FHm4 (*Fraxinus* and *Tilia*) showing similar pH values or FHm5 (*Larix* and *Pseudotsuga*) showing high Klason lignin content, especially in the sapwood network, specialization was so high that tree species were not grouped into interconnected modules.

In addition, highly modular and specialized structure of fungal interaction networks has also been observed by A. Mazziotta et al. (17) investigating fruiting bodies on deadwood. Through a comparison of fungi, bryophytes, and lichens, they concluded that the trophic relationship shapes network structures revealing mutualistic structures for both autotrophic groups and more antagonistic characteristics for the heterotrophic fungi. Their assumption, that the application of next-generation sequencing data including those fungi present just as vegetative mycelia will reveal even stronger modular community structures, has been confirmed by the present study.

Indeed, the *H_2_′* value for fungi corresponds well to that of xylophagous beetles observed for the same deadwood experiment at an early stage of decomposition (18). Interestingly, the authors of that study found a negative relationship between trophic level and specialization, the latter decreasing from wood-consuming beetles, via fungivores to predators. This observation emphasizes the high degree of specialization for xylophagous arthropods, indicating that the trophic relationship determines network properties. This, in turn, is in line with our results and reflects the strong association of fungi (compared to that of prokaryotes) to the deadwood substrates (31). Fungi are able to effectively disintegrate the lignocellulosic complex and further degrade specific polymeric deadwood resources (36, 37), but this narrow fundamental niche (2 colonized out of 13 potential resources) may also indicate higher vulnerability to disturbances such as the absence of their host species.

The role of prokaryotes in wood decomposition, in comparison to that of fungi, is rather unresolved, underinvestigated, and not well understood (25). However, due to the increasing application of next-generation sequencing techniques, knowledge about their diversity, distribution, and activity is becoming more widely available. Progressing our earlier investigations and novel findings (compare B. Hoppe et al. [38] and [39]), we here quantified the specialization of wood-colonizing prokaryotes for the first time and observed modular and specialized networks (specialization values higher than null model results based on Vazquez’s and Patefield’s approaches). This demonstrates their significant colonization ability of this habitat indicating their participating role, directly or indirectly, in the decomposition process (hypothesis 2). Nevertheless, prokaryotic interaction networks were built by a higher number of OTUs than the fungal counterparts. Consequently, networks were much more diverse and more evenly distributed, which was reflected in the higher number of deadwood partners, i.e., higher Shannon diversity, higher interaction evenness, and higher generality of trees and OTUs. But supporting our hypothesis, the matrix size-independent estimators, modularity, and degree of specialization (*H_2_*′) also clearly emphasize that prokaryotic networks were significantly less specialized than those of fungi. Prokaryotes are probably intermediary decomposers, mainly utilizing polysaccharide fragments and other residues incipiently provided by fungi. However, this does not rule out the possibility that prokaryotes degrade such compounds in a more efficient manner than fungi can accomplish.

In regard to our hypothesis 3, the measured indices of sapwood and heartwood networks were not as strongly differentiated as expected. Indeed, the number of hosts was similar in both wood compartments for fungi. Taking into account the interaction strength, fungi, like prokaryotes, showed slightly higher diversity of interactions in the sapwood. The two groups revealed different results for specialization. While fungal interactions tended to be more distinct in the sapwood, prokaryotes were more specialized in the inner part of the wood. Nevertheless, network structure differed greatly between wood compartments, as reflected by the differences within the derived modules (i.e., tree members and associated OTUs). For instance, while the fungal sapwood network was divided into 11 modules, the respective heartwood network revealed only 8 modules. Although the prokaryotic networks resulted in 6 modules each, tree species were not grouped into the same modules. For instance, while in sapwood *Populus* was a part of a bigger interconnected module (prokaryotic sapwood module 1 [PSm1]: *Acer*, *Fraxinus*, *Populus*, *Tilia*), in heartwood *Populus* formed its own module. This module comprised many OTUs of *Firmicutes* and one highly dominant OTU (22% of sequences) of *Euryarchaeota* (*Methanobacteria*) that was almost completely lacking in the other tree species and respective compartments (30), likely due to their preferred growth in pH-neutral conditions (40, 41). In accordance therewith, this tree species revealed the highest *d′* value for the prokaryotic heartwood network. Our results suggest distinct interactions in both wood compartments, probably due to specific resource conditions.

Analyzing network structures allowed for the direct comparison of two biotic groups interacting with the same deadwood resources. In the course of this, the specialization at the network level summarized the specialization of all species (i.e., OTUs). Hence, these entire communities express a gradient in specialization, including species that belong to different guilds, and not all organisms directly contribute to the decomposition process. Nevertheless, the present study has revealed highly modular and specialized interaction networks for both groups of organisms, indicating that many fungi and prokaryotes are, as expected, resource-specific colonizers. As fungi and prokaryotes share the same habitat, they inevitably interact with each other (25). Knowledge about these interactions is rather rare, but evidence exists for links between N-fixing bacteria and fungi (38, 42) or for fungal manipulation on prokaryotic growth (43, 44). Although it seems obvious that colonization of fungal and prokaryotic species and thus the topology of networks are affected by their interactions, this was beyond the scope of the current analysis. Our results, however, reveal limited host range and thus high host selectivity by fungi, whereas prokaryotes seem to colonize the deadwood substrate less selectively. Hence, the observed network patterns emphasize the strong association between fungi and their host trees, reflecting their main role in the exploitation of this resource. We are aware that the results presented here, though considering a variety of deadwood substrates, represent a case study for a single forest site at an early to middle stage of decomposition. Future studies should include different successional stages, varied forest management types, and/or varied forest biomes to test whether these interaction properties change with increasing decomposition or depend rather on the surrounding extrinsic conditions. In conclusion, the present study appears to illustrate that the application of bipartite interaction networks, based on amplicon sequencing data, is a useful tool to explore, quantify, and compare the deadwood colonizers’ relationships in various organismic groups.

## MATERIALS AND METHODS

The present study analyzes data from J. Moll et al. (30) and S. Leonhardt et al. (29), in which all details of the sampling and laboratory procedures can be found.

### Study area and sampling.

In late 2008, an experimental platform for observing deadwood decomposition was established on forest plots of the German Biodiversity Exploratories (45) and named the BELongDead (Biodiversity Exploratories Long-term Deadwood) experiment. The experimental design was introduced in more detail by T. Kahl et al. (28). Briefly, freshly cut logs of 13 temperate tree species (nine broadleaved species, namely, *Acer* spp., *Betula* spp., Carpinus betulus, Fagus sylvatica, Fraxinus excelsior, *Populus* spp., Prunus avium, *Quercus* spp., and *Tilia* spp.; and four conifers, namely, Larix decidua, Picea abies, Pinus sylvestris, and Pseudotsuga menziesii) were placed, three replicates of each, in representative research plots, each 100 by 100 m, to investigate their decomposition over the long term. Within the research plots, the 13 logs (approximately 4 m in length and with a mean diameter of 30 to 40 cm) were placed in random order beside each other with a distance of ca. 1 m.

In order to investigate the spatial distribution of wood-inhabiting communities between the heartwood and sapwood, three experimental plots with Fagus sylvatica as dominant tree species and standardized forest management practices (selection cutting) with a distance of 0.3 to 27 km at the Hainich National Park in Central Germany (latitude 51.08, longitude 10.43) were chosen and sampled in June 2014. After more than 5 years of exposition, the majority of logs have been observed to reach transition from the early to middle stage of decomposition. Bark was partly absent, but the wood largely maintained its structure and color.

Distinguishable sapwood and heartwood samples were collected as wood chips by driving an auger horizontally to the center of each of the selected logs (compare L. Noll et al. [32]). After bark removal, sapwood was collected by means of initial drilling followed by drilling for a second time to collect heartwood. In this study, the outer 5 cm of the wood was defined as sapwood and the inner part as heartwood for all tree species, keeping in mind that only Fraxinus excelsior, Prunus avium, *Quercus* spp., Larix decidua, Pinus sylvestris, and Pseudotsuga menziesii contain distinct, visible heartwood in the stricter sense. The respective terms were used synonymously for the different wood compartments: (i) that is not involved in physiological processes in the living tree (heartwood) and (ii) that carries water and nutrients vertically from root to leaves (sapwood). We hence anticipated different wood physicochemical and physiological properties as demonstrated in (46).

### DNA extraction, PCR, and sequencing.

Total community DNA was isolated from 0.25 g of each homogenized wood sample using a ZR Soil Microbe DNA MiniPrep kit (Zymo Research, Irvine, CA, USA) according to the manufacturer’s protocol. Fungal ITS2 was amplified using the primer mix P7-3N-fITS7 and P7-4N-fITS7 (forward) together with P5-5N-ITS4 and P5-6N-ITS4 (reverse) modified after K. Ihrmark et al. (47). The prokaryotic partial 16S rRNA gene was amplified using the primer mix P5-8N-515F and P5-7N-515F (forward) together with P7-2N-806r and P7-1N-806r (reverse) modified after J. G. Caporaso et al. (48). In both cases, P5 and P7 are the Illumina adapter sequences and N is the number of random nucleotides included between the target primer and Illumina adapter to increase the diversity of generated amplicons and thus the quality of sequencing results. PCR was performed in 25-μl triplicate reactions, containing 12.5 μl of GoTaq Green Mastermix (Promega, Madison, USA), 25 μM concentrations of each primer, and approximately 20 ng template DNA. The thermal profile was as follows: fungal ITS2 was amplified with a denaturation period of 5 min at 95°C followed by 33 cycles of 95°C for 1 min, 55°C for 1 min, 72°C for 1 min 15 s, and a final elongation step at 72°C for 10 min. The prokaryotic 16S rRNA gene region was amplified with a denaturation period of 3 min at 94°C followed by 32 cycles of 94°C for 45 s, 50°C for 1 min, 72°C for 1 min 30 s, and a final elongation step at 72°C for 10 min. Amplicons were sequenced with an Illumina MiSeq at the Deep Sequencing Group of the Technische Universität Dresden.

### Bioinformatics.

Raw sequence data were imported and processed using Geneious R9 (57). First, all forward and reverse reads were 5′ trimmed and adapter regions were excluded. Then, forward and reverse reads were paired and the primer sequence was excluded. Further, the paired sequences were quality trimmed using BBDuk (settings: trim low quality, minimum quality = 13) and merged to gather the full length of the fungal ITS2 gene region and of the V4 region of the prokaryotic 16S rRNA gene using BBMerge (merge rate settings: very high) from BBTools (49). Generated sequences 220 to 440 bp long for ITS2 and 220 to 280 bp for 16S rRNA genes were exported for further analysis in the pipeline SEED (50). Clustering and chimera removal were performed using USEARCH 8.1.1861 (32 bit) (51). OTU separation was based on 3% sequence dissimilarity. Fungal and prokaryotic OTUs were taxonomically assigned using the Bayesian Classifier implemented in mothur (52) against the UNITE database (version 8.0) and the SILVA database (version 138, SSURef NR99), respectively.

### Wood traits.

Wood physicochemical properties, pH, Klason lignin, acid-soluble lignin, and water content were measured and analyzed as described by J. Moll et al. (30), and the carbon (C) content was analyzed as described by L. Noll et al. (46).

### Network analyses.

Four OTU tables for fungi and prokaryotes in sapwood and heartwood were prepared by rarefaction for the network analyses to represent equal proportions of the community. As rarefaction depth may influence network structure, different rarefaction levels (deeper and shallower sampling than the reported results) were compared to ensure that all observed trends were independent of rarefaction depth at the chosen level. This level represented 29% of the ACE (abundance-based coverage estimator) estimated community richness (53). To make inferences robust against sampling effects, 1,000 different rarefied versions were produced for each OTU table and all following analytical steps were performed independently on the 1,000 versions.

To build bipartite networks, the median of the relative OTU abundances in the three deadwood replicates was calculated and reshaped into networks using the bipartite package in R (5). This approach was chosen to make the network robust to differences in the different deadwood replicates. In order to compare the inferred network topologies to random community assemblies under different constraints, for each network three complementary null models were built using Patefield’s algorithm, the swap algorithm (54), and the shuffle approach in the bipartite package (all implemented in C. F. Dormann et al. [5]). While Patefield’s algorithm maintains the original abundance distribution (marginal sums) but not the numbers of links, the shuffle algorithm maintains connectance but strongly changes abundance distributions. Finally, Vazquez’s algorithm keeps the original number of interactions and takes abundances into account in the placement of those links, without maintaining exact abundances.

Network topologies were analyzed using the functionalities of the bipartite package. The following topological characteristics were examined.

• Shannon diversity of network entries
• interaction evenness (Shannon’s evenness of network entries; higher values indicate higher evenness)
• generality of trees is equal to e^weighted mean Shannon diversity^ (weighted mean effective number of associated OTUs per tree species, higher values indicate more general interactions)
• generality of OTUs is equal to e^weighted mean Shannon diversity^ (weighted mean effective number of associated trees per fungal or prokaryotic OTU, higher values indicate more general interactions)
• modularity (bipartite algorithm of Newman’s modularity [5, 55], with 0 indicating no modularity and 1 indicating perfect modules)
• *H_2_′* based on the deviation of a species’ realized number of interactions and that expected from each species’ total number of interactions (7) (with 0 indicating no specialization and 1 meaning perfect specialization for given interaction totals)
• *d′* species-level specialization, normalized Kullback-Leibler distance ranging from 0 for generalized to 1 for perfectly specialized species (7)

All values reported represent the mean of the 1,000 rarefied versions. The variability around the mean is given in Table S1. Modules were extracted using the computeModules function (5). The modules with the most support in the 1,000 rarefied versions are reported together with the numbers and identities of the OTUs that were present in the respective modules in all analyses (Tables S2 to S5). To ensure that the reported results are robust to changes in data preparation, all analyses were also performed on OTU tables without singletons, doubletons, and tripletons. In addition, analyses of networks representing each of the three deadwood replicate sites were performed. In order to test differences between the topologies of (i) observed networks and null models, (ii) fungal and prokaryotic networks, and (iii) sapwood and heartwood networks within the groups of organisms, paired *t* tests were performed. Networks were visualized using the R package igraph (56). Module-wise medians of wood traits were plotted using the radarchart function of the fmsb package in R.

10.1128/mSphere.00856-20.2 TABLE S1 Variability around the mean (variance, standard deviation, minimum, median, maximum) of the 1,000 rarefied network versions for all estimated indices. Download Table S1, DOCX file, 0.02 MB. Copyright © 2021 Moll et al. 2021 Moll et al. This content is distributed under the terms of the Creative Commons Attribution 4.0 International license.

10.1128/mSphere.00856-20.3 TABLE S2 Module-associated trees and OTUs and their relative abundances and identities for the fungal sapwood network. Module, name of the module; present, proportion of network version that the OTU is present in (1 = 1000/1000); inBestModule, proportion of the network versions where the OTU is associated with the respective module; percInBestModule, proportion of the network versions where the OTU is associated with the respective module provided that the OTU is part of the network (= inBestModule/present); meanAbundanceModuleSamples, mean relative abundance of the OTU in all samples belonging to the module; meanAbundanceOtherSamples, mean relative abundance of the OTU in all other samples; relAbundanceModuleSamplesVsOthers, meanAbundanceModuleSamples/meanAbundanceOtherSamples. Download Table S2, XLSX file, 0.07 MB. Copyright © 2021 Moll et al. 2021 Moll et al. This content is distributed under the terms of the Creative Commons Attribution 4.0 International license.

10.1128/mSphere.00856-20.4 TABLE S3 Module-associated trees and OTUs and their relative abundances and identities for the fungal heartwood network. Module, name of the module; present, proportion of network version that the OTU is present in (1 = 1000/1000); inBestModule, proportion of the network versions where the OTU is associated with the respective module; percInBestModule, proportion of the network versions where the OTU is associated with the respective module provided that the OTU is part of the network (= inBestModule/present); meanAbundanceModuleSamples, mean relative abundance of the OTU in all samples belonging to the module; meanAbundanceOtherSamples, mean relative abundance of the OTU in all other samples; relAbundanceModuleSamplesVsOthers, meanAbundanceModuleSamples/meanAbundanceOtherSamples. Download Table S3, XLSX file, 0.07 MB. Copyright © 2021 Moll et al. 2021 Moll et al. This content is distributed under the terms of the Creative Commons Attribution 4.0 International license.

10.1128/mSphere.00856-20.5 TABLE S4 Module-associated trees and OTUs and their relative abundances and identities for the prokaryotic sapwood network. Module, name of the module; present, proportion of network version that the OTU is present in (1 = 1000/1000); inBestModule, proportion of the network versions where the OTU is associated with the respective module; percInBestModule, proportion of the network versions where the OTU is associated with the respective module provided that the OTU is part of the network (= inBestModule/present); meanAbundanceModuleSamples, mean relative abundance of the OTU in all samples belonging to the module; meanAbundanceOtherSamples, mean relative abundance of the OTU in all other samples; relAbundanceModuleSamplesVsOthers, meanAbundanceModuleSamples/meanAbundanceOtherSamples. Download Table S4, XLSX file, 0.5 MB. Copyright © 2021 Moll et al. 2021 Moll et al. This content is distributed under the terms of the Creative Commons Attribution 4.0 International license.

10.1128/mSphere.00856-20.6 TABLE S5 Module-associated trees and OTUs and their relative abundances and identities for the prokaryotic heartwood network. Module, name of the module; present, proportion of network version that the OTU is present in (1 = 1000/1000); inBestModule, proportion of the network versions where the OTU is associated with the respective module; percInBestModule, proportion of the network versions where the OTU is associated with the respective module provided that the OTU is part of the network (= inBestModule/present); meanAbundanceModuleSamples, mean relative abundance of the OTU in all samples belonging to the module; meanAbundanceOtherSamples, mean relative abundance of the OTU in all other samples; relAbundanceModuleSamplesVsOthers, meanAbundanceModuleSamples/meanAbundanceOtherSamples. Download Table S5, XLSX file, 0.4 MB. Copyright © 2021 Moll et al. 2021 Moll et al. This content is distributed under the terms of the Creative Commons Attribution 4.0 International license.

10.1128/mSphere.00856-20.7 TABLE S6 Results (*P* values) on paired *t* tests between the observed networks and respective null models for all estimated network indices. ns, not significant. Download Table S6, DOCX file, 0.01 MB. Copyright © 2021 Moll et al. 2021 Moll et al. This content is distributed under the terms of the Creative Commons Attribution 4.0 International license.

10.1128/mSphere.00856-20.8 TABLE S7 Results (*P* values) on paired *t* tests based on plot-wise networks for all estimated network indices. ns, not significant. Download Table S7, DOCX file, 0.01 MB. Copyright © 2021 Moll et al. 2021 Moll et al. This content is distributed under the terms of the Creative Commons Attribution 4.0 International license.

10.1128/mSphere.00856-20.9 TABLE S8 Species specialization index *d′* of each deadwood resource for fungal and prokaryotic sapwood and heartwood networks. Download Table S8, DOCX file, 0.01 MB. Copyright © 2021 Moll et al. 2021 Moll et al. This content is distributed under the terms of the Creative Commons Attribution 4.0 International license.

10.1128/mSphere.00856-20.10 TABLE S9 Results (*P* values) on paired *t* tests for species-level specialization *d′*. ns, not significant. Download Table S9, DOCX file, 0.01 MB. Copyright © 2021 Moll et al. 2021 Moll et al. This content is distributed under the terms of the Creative Commons Attribution 4.0 International license.

### Data availability.

All R scripts and related explanations to reproduce the network analyses are available at https://git.ufz.de/metaOmics/Deadwood-networks. All processed and merged OTU sequences have been submitted to the NCBI short read archive (SRA, https://www.ncbi.nlm.nih.gov/sra/) and are accessible under the number SRP102646.
