# Supplementary material for: Amplicon Sequencing-Based Bipartite Network Analysis Confirms a High Degree of Specialization and Modularity for Fungi and Prokaryotes in Deadwood
Source: mSphere. 2021 Jan 13;6(1):e00856-20. doi: 10.1128/mSphere.00856-20 (PMC7845612; doi:10.1128/mSphere.00856-20)
Supplement: TABLE S1 [file mSphere.00856-20_st001.docx]

| network | measure | mean | var | sd | min | med | max |
| --- | --- | --- | --- | --- | --- | --- | --- |
| prokaryotes - sap | OTUs | 1412 | 321 | 18 | 1343 | 1412 | 1480 |
| prokaryotes - heart | OTUs | 1097 | 254 | 16 | 1040 | 1097 | 1151 |
| fungi - sap | OTUs | 233 | 42 | 6 | 213 | 232 | 252 |
| fungi - heart | OTUs | 207 | 41 | 6 | 187 | 207 | 236 |
| prokaryotes - sap | Generality (trees) | 98 | 0.86 | 0.93 | 95 | 98 | 101 |
| prokaryotes - heart | Generality (trees) | 55.1 | 3.1E-01 | 0.55 | 53.4 | 55.1 | 56.9 |
| fungi - sap | Generality (trees) | 5.63 | 6.8E-03 | 0.083 | 5.38 | 5.63 | 5.88 |
| fungi - heart | Generality (trees) | 5.04 | 3.9E-03 | 0.063 | 4.81 | 5.04 | 5.23 |
| prokaryotes - sap | Generality (OTUs) | 6.58 | 8.6E-04 | 2.9E-02 | 6.49 | 6.58 | 6.67 |
| prokaryotes - heart | Generality (OTUs) | 6.77 | 9.1E-04 | 0.03 | 6.68 | 6.77 | 6.86 |
| fungi - sap | Generality (OTUs) | 2.08 | 4.3E-04 | 0.02 | 2.03 | 2.09 | 2.15 |
| fungi - heart | Generality (OTUs) | 2.12 | 2.9E-04 | 0.017 | 2.06 | 2.12 | 2.19 |
| prokaryotes - sap | Shannon | 7.01 | 8.5E-05 | 9.2E-03 | 6.98 | 7.01 | 7.04 |
| prokaryotes - heart | Shannon | 6.40 | 9.1E-05 | 9.5E-03 | 6.37 | 6.40 | 6.43 |
| fungi - sap | Shannon | 3.89 | 1.7E-04 | 1.3E-02 | 3.85 | 3.89 | 3.93 |
| fungi - heart | Shannon | 3.69 | 1.2E-04 | 1.1E-02 | 3.65 | 3.69 | 3.73 |
| prokaryotes - sap | interaction evenness | 0.714 | 1.2E-06 | 1.1E-03 | 0.71 | 0.71 | 0.72 |
| prokaryotes - heart | interaction evenness | 0.669 | 1.4E-06 | 1.2E-03 | 0.67 | 0.67 | 0.67 |
| fungi - sap | interaction evenness | 0.485 | 4.2E-06 | 2.0E-03 | 0.48 | 0.49 | 0.49 |
| fungi - heart | interaction evenness | 0.468 | 3.8E-06 | 2.0E-03 | 0.46 | 0.47 | 0.47 |
| prokaryotes - sap | H2’ | 0.313 | 3.6E-06 | 1.9E-03 | 0.31 | 0.31 | 0.32 |
| prokaryotes - heart | H2’ | 0.352 | 3.9E-06 | 2.0E-03 | 0.35 | 0.35 | 0.36 |
| fungi - sap | H2’ | 0.784 | 8.2E-06 | 2.9E-03 | 0.77 | 0.78 | 0.79 |
| fungi - heart | H2’ | 0.742 | 1.2E-05 | 3.5E-03 | 0.73 | 0.74 | 0.75 |
| prokaryotes - sap | modularity | 0.329 | 4.7E-05 | 6.8E-03 | 0.31 | 0.33 | 0.35 |
| prokaryotes - heart | modularity | 0.403 | 1.3E-05 | 3.5E-03 | 0.39 | 0.40 | 0.41 |
| fungi - sap | modularity | 0.732 | 9.7E-06 | 3.1E-03 | 0.72 | 0.73 | 0.75 |
| fungi - heart | modularity | 0.663 | 5.2E-05 | 7.2E-03 | 0.64 | 0.66 | 0.69 |
