# Supplementary material for: Amplicon Sequencing-Based Bipartite Network Analysis Confirms a High Degree of Specialization and Modularity for Fungi and Prokaryotes in Deadwood
Source: mSphere. 2021 Jan 13;6(1):e00856-20. doi: 10.1128/mSphere.00856-20 (PMC7845612; doi:10.1128/mSphere.00856-20)
Supplement: TABLE S6 [file mSphere.00856-20_st006.docx]

| Related network | Null model | Shannon | Interaction evenness | Generality (trees) | Generality (OTUs) | H2' | Modularity |
| --- | --- | --- | --- | --- | --- | --- | --- |
| Sapwood prokaryotes | Patefield | < 0.001 | < 0.001 | < 0.001 | < 0.001 | < 0.001 | < 0.001 |
|  | Vaznull | < 0.001 | < 0.001 | < 0.001 | < 0.001 | < 0.001 | < 0.001 |
|  | shuffle | ns* | ns* | < 0.001 | < 0.001 | < 0.001 | < 0.001 |
| Heartwood prokrayotes | Patefield | < 0.001 | < 0.001 | < 0.001 | < 0.001 | < 0.001 | < 0.001 |
|  | Vaznull | < 0.001 | < 0.001 | < 0.001 | < 0.001 | < 0.001 | < 0.001 |
|  | shuffle | ns* | ns* | < 0.001 | < 0.001 | < 0.001 | < 0.001 |
| Sapwood fungi | Patefield | < 0.001 | < 0.001 | < 0.001 | < 0.001 | < 0.001 | < 0.001 |
|  | Vaznull | < 0.001 | < 0.001 | < 0.001 | < 0.001 | < 0.001 | < 0.001 |
|  | shuffle | ns* | ns* | < 0.001 | < 0.001 | < 0.001 | < 0.001 |
| Heartwood fungi | Patefield | < 0.001 | < 0.001 | < 0.001 | < 0.001 | < 0.001 | < 0.001 |
|  | Vaznull | < 0.001 | < 0.001 | < 0.001 | < 0.001 | < 0.001 | < 0.001 |
|  | shuffle | ns* | ns* | < 0.001 | < 0.001 | < 0.001 | < 0.001 |

* not meaningful for these diversity related indices as no differences are expected because the Nullmodel’s connectance has to be equal to the observed data
