# Supplementary material for: Amplicon Sequencing-Based Bipartite Network Analysis Confirms a High Degree of Specialization and Modularity for Fungi and Prokaryotes in Deadwood
Source: mSphere. 2021 Jan 13;6(1):e00856-20. doi: 10.1128/mSphere.00856-20 (PMC7845612; doi:10.1128/mSphere.00856-20)
Supplement: TABLE S7 [file mSphere.00856-20_st007.docx]

| Groups | | Shannon | Interaction evenness | Generality (trees) | Generality (OTUs) | H2' | Modularity |
| --- | --- | --- | --- | --- | --- | --- | --- |
| Fungi vs Prokaryotes | Sapwood | 0.003 | 0.005 | 0.004 | 0.0006 | 0.005 | 0.005 |
|  | Heartwood | 0.0002 | 0.002 | 0.01 | 0.01 | 0.02 | 0.02 |
| Sapwood vs heartwood | Prokaryotes | ns | ns | 0.01 | ns | ns | ns |
|  | Fungi | ns | ns | ns | ns | ns | ns |
