# Supplementary material for: Amplicon Sequencing-Based Bipartite Network Analysis Confirms a High Degree of Specialization and Modularity for Fungi and Prokaryotes in Deadwood
Source: mSphere. 2021 Jan 13;6(1):e00856-20. doi: 10.1128/mSphere.00856-20 (PMC7845612; doi:10.1128/mSphere.00856-20)
Supplement: TABLE S8 [file mSphere.00856-20_st008.docx]

|  | Fungi | | Prokaryotes | |
| --- | --- | --- | --- | --- |
| Deadwood resource | Sapwood | Heartwood | Sapwood | Heartwood |
| *Acer* | 0.700 | 0.844 | 0.226 | 0.212 |
| *Betula* | 0.830 | 0.706 | 0.297 | 0.644 |
| *Carpinus* | 0.610 | 0.588 | 0.224 | 0.211 |
| *Fagus* | 0.866 | 0.604 | 0.332 | 0.214 |
| *Fraxinus* | 0.894 | 0.833 | 0.397 | 0.286 |
| *Larix* | 0.494 | 0.558 | 0.204 | 0.259 |
| *Picea* | 0.844 | 0.949 | 0.179 | 0.212 |
| *Pinus* | 0.806 | 0.507 | 0.386 | 0.232 |
| *Populus* | 0.651 | 0.769 | 0.370 | 0.712 |
| *Prunus* | 0.529 | 0.624 | 0.269 | 0.229 |
| *Pseudotsuga* | 0.863 | 0.865 | 0.340 | 0.211 |
| *Quercus* | 0.708 | 0.704 | 0.182 | 0.276 |
| *Tilia* | 0.892 | 0.651 | 0.437 | 0.347 |
