# Supplementary material for: Amplicon Sequencing-Based Bipartite Network Analysis Confirms a High Degree of Specialization and Modularity for Fungi and Prokaryotes in Deadwood
Source: mSphere. 2021 Jan 13;6(1):e00856-20. doi: 10.1128/mSphere.00856-20 (PMC7845612; doi:10.1128/mSphere.00856-20)
Supplement: TABLE S9 [file mSphere.00856-20_st009.docx]

| Groups | | d‘ |
| --- | --- | --- |
| Fungi vs Prokaryotes | Sapwood | 1.00E-08 |
|  | Heartwood | 1.00E-05 |
| Sapwood vs heartwood | Prokaryotes | ns |
|  | Fungi | ns |
